# Protective Effects of the Segmental Renal Artery Clamping Technique on Ischemia-Reperfusion Injury in db/db Diabetic Mice

**DOI:** 10.1155/2017/4763828

**Published:** 2017-02-12

**Authors:** Chao Liang, Jundong Zhu, Chenkui Miao, Shangqian Wang, Lei Zhang, Pu Li, Zengjun Wang, Pengfei Shao

**Affiliations:** Department of Urology, The First Affiliated Hospital of Nanjing Medical University, Nanjing, China

## Abstract

Renal ischemia-reperfusion (I/R) injury is inevitable in partial nephrectomy and other kidney surgeries, with a higher incidence in patients with renal insufficiency. This study aimed to investigate the protective effects of precise segmental renal artery clamping (SRAC) against renal I/R injury in db/db diabetic mice, compared with conventional renal artery clamping (RAC). Grape seed extract, a powerful free radical scavenger, was administered to diabetic mice for 4 weeks before operation in subgroups (30 mg/kg/d). The unilateral renal pedicle was ligatured, and I/R injury to the contralateral kidney was induced (ischemia for 30 min followed by reperfusion for 24 h). Blood glucose value, creatinine, blood urea nitrogen, and urine microalbumin/urine creatinine ratio increased gradually and showed no preoperative statistical differences among six subgroups. These parameters were significantly lower in the SRAC than in the RAC group 24 h postoperatively. Moreover, the nonischemic area in the SRAC group expressed less KIM-1 and TNF-*α* mRNA and also revealed minor histopathological damage induced by I/R. These findings suggest that SRAC effectively reduces early renal injury induced by I/R and accelerates the recovery of renal function in diabetic mice. Thus, SRAC may be an ideal technique in partial nephrectomy, especially for patients with diabetic nephropathy and other renal insufficiencies.

## 1. Introduction

Ischemia-reperfusion (I/R) injury is a common clinical pathological and physiological phenomenon. It is a kind of cell metabolism disorder that occurs as a result of ischemia and reperfusion and leads to the destruction of structure and function. Ischemia and reperfusion can occur in many tissues and organs of the human body, such as the heart, brain, liver, kidney, lung, and gastrointestinal tract. The kidney is an organ that is prone to sustain I/R injury during partial nephrectomy and other kidney surgeries, resulting in the occurrence of acute kidney injury (AKI) [1, 2].

Attributed to hyperglycaemia, dyslipidemia, and other metabolic disorders, diabetes mellitus (DM) has become a common chronic metabolic disease, with a global prevalence of nearly 400 million patients [3]. Various clinical trials have verified DM as a susceptibility factor for the occurrence of diabetic nephropathy [4, 5]. Diabetic nephropathy has been considered a consequential cause of mortality in the diabetic population. On account of the vessel lesions induced by DM, the tolerance to I/R injury is compromised significantly and the kidney tends to develop acute renal injury more easily [6]. By clamping renal vessels for 30 min, irreversible acute kidney injury has been shown to occur in diabetic mice, when compared to a nondiabetic group [7, 8]. Therefore, it is clinically essential to investigate possible and effective therapies to ameliorate renal ischemia-reperfusion injury.

During partial nephrectomy and other renal surgeries, various approaches have been used to reduce ischemia-reperfusion injury. Utilizing different renal vascular clamping types is an efficient method to prevent ischemia-reperfusion damage and has been applied to laparoscopic partial nephrectomy and other radical surgeries [9]. Compared with complete renal artery clamping, segmental artery clamping could minimize intraoperative ischemia injury and improve early postoperative renal function [10, 11]. Furthermore, a series of animal experiments have indicated that precise renal artery clamping could promote the activity of the remaining renal unit and decrease ischemia-reperfusion injury more efficiently than complete renal pedicle blocking [12, 13].

Some biochemical and histomorphological indicators may be used to assess injury induced by renal ischemia-reperfusion, such as creatinine, blood urea nitrogen (BUN), and urine microalbumin/urine creatinine (UMAB/Ucr) ratio. These indices can be used to evaluate glomerular filtration function, among which UMAB/Ucr ratio in particular can detect early renal damage. Additionally, the morphological structure of mitochondria and histopathological changes reflecting the impairment of glomeruli induced by ischemia and reperfusion may be explored. Kidney injury molecule-1 (KIM-1), belonging to type I transmembrane glycoprotein, has also been shown to be a sensitive biomarker for acute kidney injuries. The expression level of KIM-1 was positively correlated with the degree of renal injury [14]. Tumour necrosis factor-alpha (TNF-*α*) is another worthwhile biomarker, giving rise to the extension of ischemia-reperfusion injury [15, 16]. Thus, to explore the molecular mechanism of acute renal injury, we detected the relative expression of KIM-1 and TNF-a derived from mice kidney tissues after 24 hours of reperfusion. A powerful free radical scavenger, grape seed extract (GSE), has antioxidant activity and helps to prevent oxidative damage induced by ischemia-reperfusion [17]. On the basis of different artery clamping types, the role of GSE in ischemia-reperfusion injury can be observed.

Broadly speaking, by using different surgical models, the purpose of this study was to evaluate the effect of different renal artery clamping techniques on the recovery of renal function and to determine a superior intraoperative treatment during partial nephrectomy or other kidney surgery.

## 2. Methods and Material

### 2.1. Animal Models and Groups

A total of 42 male C57BLKS/J db/db (db/db, 8 weeks old) mice were purchased from Model Animal Research Center of Nanjing University (Jiangsu, China). They were raised in light-controlled coops in an animal room and received free access to chow and tap water in a stationary periodically environment. The conditions of housing room were controlled at a fixed level with room temperature 20–25°C, room humidity 40%–60% and a 12 h light/dark cycle. All experimental procedures received approval from the animal ethics committee of Nanjing Medical University. Randomly, these 42 C57BLKS/J db/db mice were divided into six groups at the beginning of study: named GSE(−)/SRAC group (placebo + SRAC, *n* = 7); GSE(−)/RAC group (placebo + RAC, *n* = 7); GSE(−)/sham group (placebo + sham, *n* = 7); GSE(+)/SRAC group (GSE + SRAC, *n* = 7); GSE(+)/RAC group (GSE + SRAC, *n* = 7); and GSE(+)/sham group (GSE + sham, *n* = 7), respectively. Each group of mice was numbered from one to seven independently. From thirteen weeks old, the GSE-treated experimental groups of mice were treated with grape seed extracts by intragastric administration whereas the GSE(−) control group is given placebo treatment. Errhysis was not allowed when filling the stomach due to the blood soluble characteristic of GSE. Each group of mice was observed for eight weeks without any administration of intervention therapy. At the end of the treatment, all mice were observed for overnight and then sacrificed. The blood (200 *μ*l) and urine (250 *μ*l) were sampled to further detection on the second day. In addition, ligation zone and ischemic and normal areas of kidney tissues were sampled for encapsulation and kept at −80°C, glutaraldehyde for further analysis.

### 2.2. Drugs Treatment

Grape seed extracts (purity > 99.9%, Jianfeng Inc., Tianjin, China) were obtained from the wastes of grape molasses production and experienced a series of complex postprocessing. GSE-treated experimental group was given GSE daily in normal saline solution, at a dosage of 30 mg/kg, by gavage administration from thirteen weeks. The control group of mice was given equal amount of normal saline solution instead. In the meantime, additional breeding conditions of the whole six groups should keep in accordance with each other.

### 2.3. Surgical Procedure

**Figure 1:**
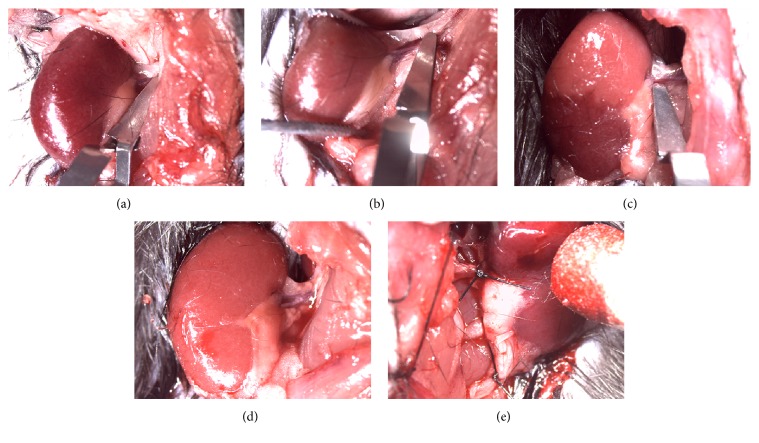
Details of different vascular clamping among surgical procedure. (a-b) Complete renal artery clamping and its ischemia-reperfusion process. (c-d) Segmental renal artery blocking and ischemia-reperfusion process. (e) Ligation of the contralateral renal pedicle and the ureter.

As is mentioned above, a total of 42 diabetic mice were assigned into six subgroups according to different operation modes, named GSE(−)/SRAC group; GSE(−)/RAC group; GSE(−)/sham group; GSE(+)/SRAC group; GSE(+)/RAC group; and GSE(+)/sham group respectively. Primarily, the mice were anaesthetized with a mixture of Xylocaine via abdomen cavity which diluted in sterile saline to a final volume of 2.5 ml/100 g body weight. The animal was immediately transferred to a constant temperature mat until loss of righting reflex has occurred. It is of foremost necessity that body temperature is monitored during anesthesia stage for it may decrease among this process. To improve working efficacy of surgery and obtain a more efficient time use, preoperative hair removal of each mouse was of prime importance during the whole surgical procedure. Next, anaesthetic depth and respiration frequency of mice were monitored, respectively, before setting out the surgery. After disinfection, the abdomen was opened along the linea alba incision with a length approximately 1.0–1.5 cm. After disinfection, the abdomen was opened along the linea alba incision with a length approximately 1.0–1.5 cm, then pushing the intestines aside carefully and making the kidney exposed sufficiently and using suitable size tweezers to remove the surrounding adipose tissue and making the blood vessels exposed for renal pedicle clamping. The unilateral renal pedicle was ligatured with operation silk thread and made every effort to ensure minimized vascular damage as little as perihilar fat (Figure 1(e)). The segmental renal artery of SRAC group mice was clamped for 30 min by noninvasive vascular clip and released at a eligible time to initiate reperfusion (Figures 1(c) and 1(d)). In this process, segmental renal artery of each mouse was observed by optical microscope and clamped randomly, whereas RAC group mice experienced an identical management as the SRAC group has except for entire renal artery clamping only (Figures 1(a) and 1(b)). Of course, sham-operated mice were attached to the exact same surgical procedure, apart from clamp placement. After surgical procedure, the abdomen of each mouse was temporarily sutured and the animal was transferred to a warm incubator to be kept at a stable temperature for the duration of reperfusion. A thorough ischemia was observed when the colour of kidney changes from red to dark purple gradually. The ischemic and nonischemic regions in different groups of mice were depicted on a draft respectively. Then all mice of six subgroups were monitored for 24 h and sampled for further analysis. Based on the straightforward kidney graph we have described before, the kidney tissue was obtained from the upper pole and the inferior pole from SRAC group severally, while RAC group was picked from the upper or inferior pole at random. Blood sample of tail vein and urine sample were gathered from each mouse, respectively. Eventually, animals were euthanized and disposed properly by using intravenous pentobarbital.

### 2.4. Estimation of Relevant Data

All mice were weighed weekly before intragastric administration from eight weeks to sixteen weeks. Blood glucose was measured every two weeks for ensuring the hyperglycemia models of mice. Creatinine of serum and urine were measured every two weeks using creatinine assay kit (Wako, Japan), by an automatic biochemical analyzer (Hitachi 7200, Japan). In the similar way, BUN was detected as aforesaid using a BUN assay kit (Wako, Japan). Urine albumin excretion was measured by competitive enzyme-linked immunosorbent assay (ELISA) biweekly [18]. The urine of all mice was obtained by metabolic coops.

### 2.5. Histological Examination

Different poles and areas of kidneys were fetched 24 h after surgery for histological examination by light and electron microscopy. 4% paraformaldehyde-fixed tissues were embedded in paraffin, sectioned at 5 *μ*m, and stained with hematoxylin-eosin for general histological examination with a light microscope (Nikon, Tokyo, Japan) [19]. Ten glomeruli were randomly selected in the three sections from each tissue and the morphology of glomeruli with mesangial expansion was evaluated by a histopathologist blind to treatment groups. The glomerular area was also traced along the outline of the capillary loop using computer-assisted colour image analyzer LUZEX F (Nikon, Tokyo, Japan). For ultrastructural evaluation, tissues from different groups were fixed in 3% glutaraldehyde in 0.1 M cacodylate buffer solution (pH 7.4) at 4°C for 4 h and subsequently fixated in 1% osmium tetroxide phosphate buffer solution for 1 h. Next, they were dehydrated in a graded ethanol series with acetone, permeated, and embedded in epoxide resin. Ultrathin sections were stained with uranylacetate and lead citrate and examined under an H-800 electron microscope (TEM, Hitachi Electronic Instruments, Tokyo, Japan).

### 2.6. RNA Isolation and qRT-PCR

Total RNA was isolated from a section of upper/inferior pole (sham and RAC group) and ischemic/nonischemic area (SRAC group) using Trizol (Invitrogen, USA). RNA concentration and purity were measured by NanoDrop (Thermo Scientific, USA). cDNA was converted from total RNA using high capacity RNA-to-cDNA kit (Applied Biosystems, USA) according to the manufacturer's protocol. The cDNA of KIM-1 and TNF-*α* were analyzed by ABI 7300 Real-Time PCR System (Applied Biosystems, USA). The primers were designed as follows: KIM-1, 5′-ACATATCGTGGAATCACAACGAC-3′, 5′-ACTGCTCTTCTGATAGGTGACA-3′; TNF-*α*, 5′-CAGGCGGTGCCTATGTCTC-3′, 5′-CGATCACCCCGAAGTTCAGTAG-3′; *β*-actin, 5′-TCATGAAGTGTGACGTTGACATCCGT-3′, 5′-CCTAGAAGCATTTGCGGTGCACGATG-3′. Amplification was performed under the following conditions: 50°C for 2 min; 95°C for 2 min; 40 cycles at 95°C for 15 s; 60°C for 1 min; 95°C for 15 s; 60°C for 1 min; 95°C for 15 s. Each reaction replicated three times and the expression was normalized to the reference gene *β*-actin. Relative expression of KIM-1 and TNF-*α* was calculated using the 2^−ΔCt^ method.

### 2.7. Statistical Analyses

Data were expressed as mean ± SD. Statistical comparisons between the groups were analyzed using Shapiro-Wilk test and Student's *t*-test. All the statistical analyses were carried out using SPSS (SPSS for Version 20). *p* value < 0.05 was considered statistically significant.

## 3. Results

### 3.1. Body Weights and Blood Glucose Value

Mean body weights and blood glucose values in six groups of mice are listed in detail in Table 1. At the beginning of the study, all experimental mice had similar baseline body weights and almost equivalent blood glucose levels. After one week of GSE treatment, glucose levels did not show a significant difference from those at the onset, and there was no statistically significant difference among the six subgroups (*p* > 0.05). However, mean body weight displayed a slowly increasing trend along the whole feeding process. After performing the surgical procedure at sixteen weeks, both glucose levels and body weights were significantly elevated than before across all six subgroups. However, differences among the six subgroups of mice failed to achieve statistical significance (*p* > 0.05, data not shown).

### 3.2. Serum Creatinine, BUN, and UMAB/Ucr Levels

**Figure 2:**
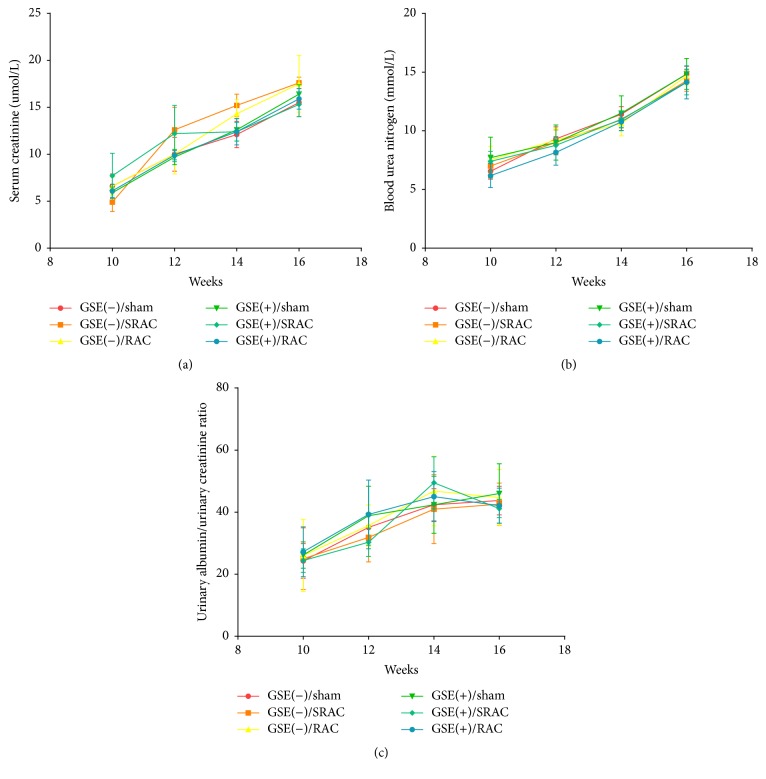
Creatinine, blood urea nitrogen (BUN), and UMAB/Cr levels of six groups mice before surgical procedure. (a-b) Creatinine and BUN levels of blood increased slowly during the investigation, but they showed no statistical difference in six groups. (c) UMAB/Cr values increased progressively during the 8-week observation period, but there were no significantly differences between the groups. Data are mean ± SEM.

Before clamping the renal artery, serum creatinine and BUN levels did not show significant differences in the six subgroups during our investigation period (Figures 2(a) and 2(b)). The UMAB (urine microalbumin), which served as a characteristic feature of diabetic nephropathy, increased progressively in all six groups. Nevertheless, there were no significant differences in UMAB/Ucr ratio among six groups throughout the total period (Figure 2(c)). The GSE-treated experimental group was given GSE daily via intragastric administration at thirteen weeks, while the control group was not. Prior to the surgical procedure, treatment of experimental mice with GSE did not obviously inhibit increases of creatinine, BUN, and UMAB/Ucr in comparison with placebo-treated mice, which indicated a nonenhancement of renal function after GSE treatment.

**Figure 3:**
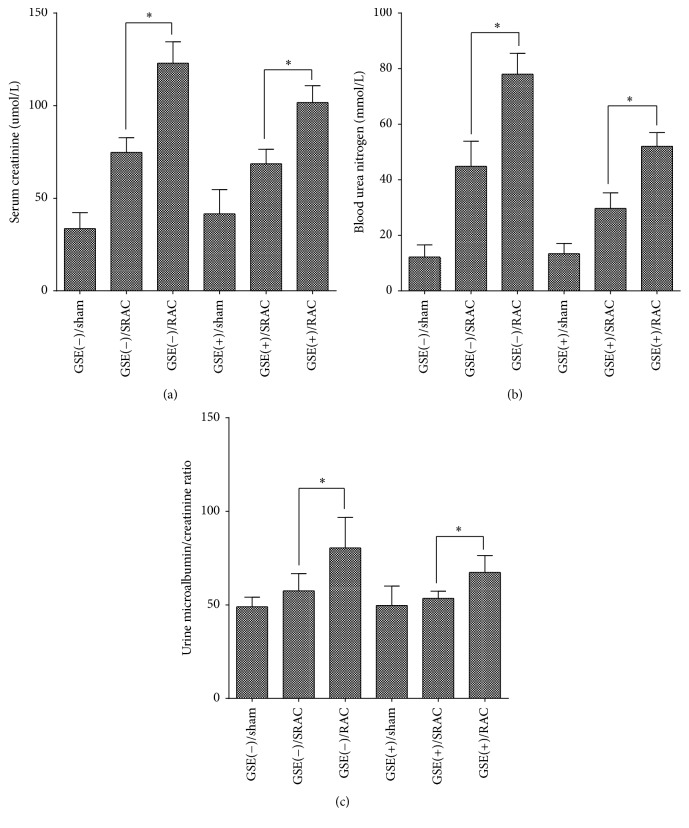
Creatinine, blood urea nitrogen (BUN), and UMAB/Cr levels of six groups mice after surgical procedure. (a-b) Creatinine and BUN levels of blood were obviously lower in SRAC groups than in RAC groups after different surgery types. No obvious difference was found between GSE(+) groups and GSE(−) groups. Data are mean ± SEM, ^*∗*^*p* < 0.05. (c) UMAB/Cr values showed similar discrepancy between SRAC and RAC group mice. Data are mean ± SEM, ^*∗*^*p* < 0.05.

However, after operation, serum creatinine and BUN levels in the GSE(+)/SRAC and GSE(−)/SRAC groups were significantly lower than those in the GSE(+)/RAC and GSE(−)/RAC groups (*p* < 0.05). Meanwhile, creatinine and BUN were lower in the GSE(+)/SRAC group than the GSE(−)/SRAC group, and the same parameters in the GSE(+)/RAC group were lower than in GSE(−)/RAC group. The GSE(+)/sham group and GSE(−)/sham group exhibited the lowest creatinine and BUN among all six groups (Figures 3(a) and 3(b)). In addition, the UMAB/Ucr levels brought out trends similar to creatinine and BUN (Figure 3(c)).

### 3.3. Minor Histopathological Damage Was Found in the Nonischemic Area of the SRAC Group

**Figure 4:**
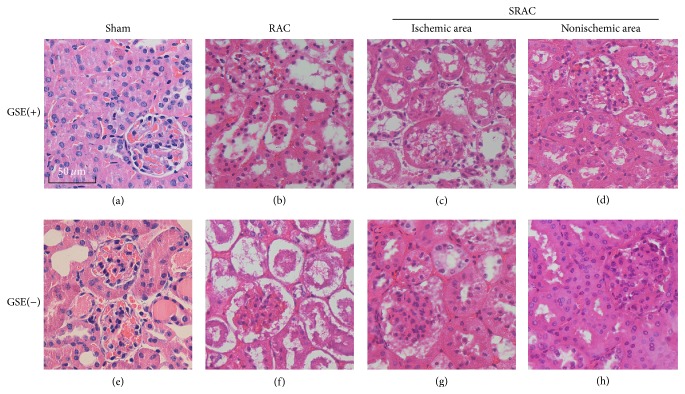
SRAC reveals protective effects on glomeruli and proximal tubule against renal I/R injury in db/db diabetic mice. Representative sections from different groups were fetched 24 h after surgery for histological examination (HE stained, magnification ×400). No obvious histological alternations are found in sham group mice (a, e). Kidneys in nonischemic area of SRAC group are characterized by mild tubular dilatation containing a few exfoliated cells (d, h), while proximal tubule cell necrosis, vacuolization, tubular dilatation, and intratubular exfoliated cells are founded in ischemic area (c, g). Kidneys from RAC group mice show the worst disrupted cytoarchitecture with cavity expansion of glomeruli, greater amount of exfoliated intratubular cells, and cylinders in proximal and collecting tubules (b, f). Given GSE or not has a similar pattern between different-treated groups in our experiment.

Histological alterations were not detected in kidneys from the sham group of mice (Figures 4(a) and 4(e)), and the kidney tissue from nonischemic areas was characterized by mild tubular dilatation containing a few exfoliated cells (Figures 4(d) and 4(h)). In addition, vacuolization and tubular dilatation were observed in ischemic areas (SRAC group). There was denudation of tubular basement membranes, together with the presence of a greater number of intratubular exfoliated cells at different stages of degeneration (Figures 4(c) and 4(g)). Kidneys from RAC group of mice showed the worst disrupted cytoarchitecture due to an even greater amount of exfoliated intratubular cells and cylinders in proximal and collecting tubules (Figures 4(b) and 4(f)). In our experiment, there was a barely significant effect of GSE and renal tissue treatment on renal cytoarchitecture between groups.

**Figure 5:**
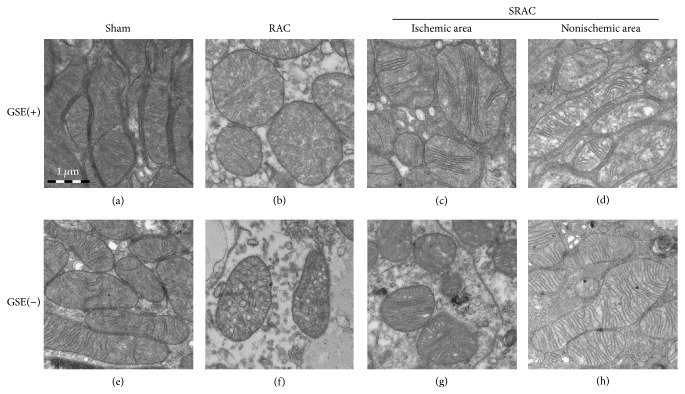
SRAC protects mitochondrial structure during renal ischemia-reperfusion. Kidney sections obtained 24 h after surgery were examined by electron microscopy (×80,000). Mitochondria in sham group mice are elongated with densely packed cristae membranes (a, e). Mitochondria in nonischemic area of SRAC group are also elongated with numerous cristae membranes, although cristae are not as densely packed as in sham group (d, h). Mitochondria from RAC group mice are grossly swollen and cristae are collapsed together with an extremely reduction of matrix density (b, f). Although most mitochondria are still rounded in ischemic area of SRAC group, they contain more cristae membranes and have more condensed matrix density than RAC group (c, g). The overall morphological structure changing of mitochondria in GSE(+) and GSE(−) group is similar besides the matrix density.

**Figure 6:**
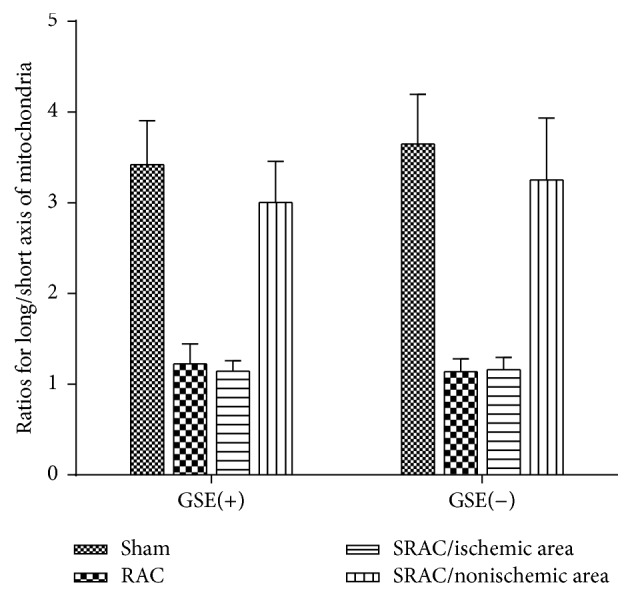
Ratios for long/short axis of mitochondria in different groups 24 h after the surgery. The size and ratios for long/short axis of 10 mitochondria randomly in each group were measured, and a histogram was made. The long axis size of mitochondria was almost 3 to 4 times as the short axis size in sham group and nonischemic area (SRAC group). However, the long/short axis ratio in RAC group and ischemic area (SRAC group) was close to 1.

We also explored the morphological structure of mitochondria between differently treated renal tissues under the electron microscope. Examination of the sham group revealed elongated mitochondria with densely packed cristae that were organized along the actin cytoskeletal structure (Figures 5(a) and 5(e)). In the SRAC/RAC groups, mitochondria underwent dramatic morphological changes after 30 min of ischemia. In addition to being large and rounded, the most commonly observed changes of mitochondria were swelling in the cristae and matrix with a loss of cristae membranes. After 30 minutes of ischemia, mitochondria in the proximal tubules of the RAC group were dramatically swollen, with a reduction of matrix density. There was almost a complete loss of cristae membranes and the remaining cristae were very short and condensed (Figures 5(b) and 5(f)). Although most mitochondria were still rounded in the ischemic area of the SRAC group, they contained more cristae membranes and had more condensed matrix density than the RAC group (Figures 5(c) and 5(g)). In contrast, mitochondria architecture was well-preserved in the nonischemic areas of the SRAC group, and there was minimal loss of matrix density while mitochondria remained elongated (Figures 5(d) and 5(h)). The overall morphological structure change of mitochondria in the GSE(+) and GSE(−) groups was similar. We then measured the size and ratios for long/short axis of 10 mitochondria randomly in each group, drawing a histogram of different groups (Figure 6).

### 3.4. Nonischemic Areas Expressed Less KIM-1 and TNF-*α* mRNA

**Figure 7:**
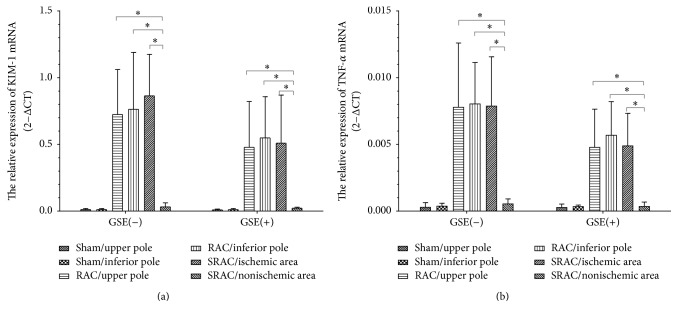
Nonischemic area expresses less KIM-1 and TNF-*α* mRNA. Kim-1 (a) and TNF-*α* (b) mRNA expression were measured by RT-PCR and 2^−ΔCt^ were calculated. KIM-1 and TNF-*α* mRNA in renal tissues rise markedly after the surgery except the mice in sham group. However, expression of KIM-1 and TNF-*α* mRNA in nonischemic area (SRAC group) was much lower than ischemic area (SRAC group) and RAC group (*p* < 0.05). Overall expression of KIM-1 and TNF-*α* mRNA in GSE(−) group was higher than GSE(+) group but this difference failed to achieve statistical significance. Data are mean ± SEM; ^*∗*^*P* < 0.05.

RT-PCR was conducted and 2^−ΔCt^ values were calculated as described in Methods. We found almost no KIM-1 while TNF-*α* mRNA was expressed in the sham group. Although KIM-1 and TNF-*α* mRNA expression increased in both RAC and SRAC groups, no statistically significant difference was found for the upper/inferior pole (RAC group) and ischemic areas (SRAC group). However, expression of KIM-1 and TNF-*α* mRNA in nonischemic areas (SRAC group) was much lower than in the former three groups (*p* < 0.05). Overall expression of KIM-1 and TNF-*α* mRNA in the GSE(−) group was higher than that in the GSE(+) group but this difference failed to achieve statistical significance (Figure 7).

## 4. Discussion

In general, the kidney is considered a hypertransfusion organ that is very sensitive to ischemia and hypoxia. Ischemia can lead to the decrease of renal blood flow, renal tubular congestion, and severe renal tubular necrosis, contributing to renal injury after reperfusion and eventual ischemic acute renal failure. At present, it is generally believed that the increase of free radicals and reactive oxygen species can bring about a large amount of malondialdehyde (MDA) and other toxic substances. Oxygen free radicals can damage lipids of biological membranes and cause lipid peroxidation, resulting in a large amount of cytotoxic lipid peroxidation MDA. A cross chain of MDA is formed in the membrane, resulting in impaired membranes, mitochondrial swelling, and dissolution of adenosine triphosphate (ATP) synthesis, lysosomal rupture, and imbalances in unsaturated fatty acid and protein levels [20]. Ultimately, oxygen free radicals cause biological membrane dysfunction along with decreased fluidity and increased permeability. After reperfusion, a series of lipid peroxidation products of kidney tissue, such as MDA, increases [21, 22]. Thus, the renal tubular epithelial cells are injured, resulting in decreased filtration function and abnormal renal function. According to current research, serum creatinine and blood nitrogen are usually measured to assess overall kidney function. Declining renal function is indicated by increasing serum urea nitrogen and creatinine values. In addition, urinary nitrogen, creatinine, and urine microalbumin increase gradually due to damage of the glomerular filtration membrane [23].

In our study, we evaluated a series of indicators, mentioned above, during the experimental period. The impaired renal function consequent to hyperglycaemia levels can be progressively supervised using serum creatinine and urea nitrogen levels. Assuredly, the plasma creatinine and nitrogen content are statistically higher in DM mice when compared with baseline values our study [24].

Along with prolonged lifespans and improvement in living standards, diabetes mellitus (DM) is a common and increasing chronic endocrine disorder mainly caused by hyperglycaemia and metabolic diseases. Diabetic patients with persistent hyperglycaemia can sustain permanent dysfunction of organs including kidneys, hearts, eyes, and nerves. Microalbumin has been positively correlated with higher glucose levels among patients with type 2 DM [24]. In accordance with this, a previous study has verified that there is a 49% morbidity rate of microalbuminuria among patients with different types of DM [25]. Recently, a novel research study detected kidney damage in patients with type 2 diabetes mellitus by comparing the microalbumin/creatinine ratio with 24-hour urinary albumin content. They concluded that microalbumin/creatinine ratio could serve as a preferential choice for evaluating the degree of early renal trauma [26]. Therefore, we identified the UMAB/Ucr ratio as a sensitive marker for early renal impairment.

In our study, we detected the level of UMAB and Ucr in all six groups of DM mice. During the whole research period, UMAB/Ucr ratio levels did not vary markedly between GSE(+) groups and GSE(−) groups. Other relevant indicators such as serum urea nitrogen and creatinine also showed no statistical significance among the six subgroups, revealing that GSE may not produce a positive effect on the recovery of kidney function in patients with diabetes mellitus. Consistent with our experimental findings, Yonguc discovered that the mean level of blood glucose and body weight were not affected significantly by GSE by comparing GSE-treated diabetic mice to normal diabetic mice. This verified that GSE did not improve the symptoms of diabetes. Nevertheless, GSE and other treatments can ameliorate the degree of oxidative stress in the hippocampus and blood of diabetic mice [17]. Consistent with our conclusions, Sato et al. have described that GSE has antioxidant activity and can be regarded as an important factor in preventing ischemia-reperfusion induced oxidative damage and apoptosis in cardiomyocytes [27].

Consistent with data from a previous essay, our study reported an experimental model of kidney ischemia in respect to temporary artery clamping [28]. In addition, the duration of renal ischemia is very vital and is often regulated at 30–60 minutes. Renal clamping duration of more than one hour may cause irreversible acute tubular necrosis and renal damage. Less than 30 minutes would promote rapid proliferation of tubular epithelial cells and repair renal tubules [29]. Thus, in our study, unilateral renal arteries were clamped to induce ischemia for 30 min followed by reperfusion for 24 hours. After the surgical procedure, the function of the kidney was evaluated by biochemical measurements (BUN, creatinine, and UMAB/Ucr), some cytokine activities (KIM-1 and TNF-*α*), haematoxylin and eosin (HE) staining, and transmission electron microscopic observations [13]. Previous studies have confirmed that precise segmental renal artery clamping can reduce the extent of renal ischemia and reperfusion injury, while the nonsurgical regions were not affected. Contrary to total renal artery clamping, segmental renal artery clamping can maintain the integrity of the cell membrane and organelle membrane of the normal nonblocked parts and reduce reoxygenation injury [30].

Our experiment found that the serum creatinine and BUN in the GSE(+)/SRAC and GSE(−)/SRAC groups were reduced significantly when compared to the GSE(+)/RAC and GSE(−)/RAC groups, which demonstrated that segmental renal artery clamping versus total renal artery clamping could ameliorate renal function in diabetic mice. Furthermore, the UMAB/Ucr ratios of the RAC groups were elevated significantly compared to SRAC groups after the surgical processes. As previously described, UMAB/Ucr ratio is usually regarded as a sign to estimate the degree of early nephropathy damage. As a consequence, our research suggested that segmental renal vascular clamping could effectively reduce early renal injury and accelerate the recovery of renal function in diabetic mice when compared to complete clamping. Precise segmental renal artery clamping during partial nephrectomy is a rational approach to preserve the remaining kidney unit when compared to the total artery blocking.

The histology showed obvious discrepancies between the severity of tissue injury and the surgical approach taken. Dramatic cellular ATP depletion is observed within 5–10 min of renal ischemia in a previous study [31] and ATP depletion influences the cytoskeletal organization as well as ATP-dependent ion transport. ATP is required for actin polymerization, and a rapid drop in ATP leads to a breakdown of the brush border, loss of cell-cell contact, disruption of barrier function, and cell detachment in proximal tubules [32–34]. The enzyme Na^+^-K^+^-ATPase plays a key role in electrolyte transport and fluid regulation in the kidney. The kidney is rich in Na^+^-K^+^-ATPase, especially the medullary thick ascending limb. In ischemia, the concentration of intracellular Na^+^ can increase by 3 or 4 times due to the inhibition of Na^+^-K^+^-ATPase activity, resulting in cell swelling [34–36]. Subsequently, the increased osmotic gradient drives water into the mitochondrial matrix through the Na^+^/H^+^ exchanger and causes matrix density reduction, cristae swelling, and the loss of cristae membranes [37, 38]. Mitochondrial function is vital to the recovery of ATP because mitochondria have minimal glycolytic capacity and must rely on oxidative phosphorylation for ATP synthesis. However, thirty minutes of renal ischemia in mice causes unfolding or loss of cristae membranes and inhibits mitochondrial respiration [34]. Therefore, the recovery of ATP synthesis upon reperfusion is delayed and a series of injuries to renal function are formed [39].

Kidney injury molecule-1 (KIM-1) is a type I transmembrane glycoprotein mainly expressed inside the kidney cortex, proximal tubular epithelial cells, and interstitial substance of exterior medullary mass, especially the S3 segment of proximal tubule of exterior medullary mass [40, 41]. KIM-1 has been shown to be a sensitive biomarker for the detection of various kidney injuries induced by drugs [42], toxins [43], and ischemia [44], as well as early stages of repair. Peng et al. demonstrated that KIM-1 in rat expression was the most sensitive in urine compared with other ischemic acute kidney injury markers such as monocyte chemoattractant protein-l (MCP-1) and cystatin C (Cys C) [40]. Meanwhile, KIM-1 in serum and urine rose with the extension of reperfusion time and reached a peak 24 hours after reperfusion. There was another study that showed that KIM-1 expression continued to be higher at 120 hours after ischemia-reperfusion (I/R) [45]. TNF-*α* is considered one of the earliest proinflammatory cytokines after trauma or infection and helps to propagate the extension of I/R injury [15, 16]. Previous researchers found that the expression of TNF-*α* in early renal I/R tissue increased significantly, which indicates that TNF-*α* might participate in early renal injury [46]. Various evidence has clearly demonstrated that TNF-*α* modulates the activation of nuclear factor kappa B (NF-kB) [47], which plays an important role in the regulation of many proinflammatory genes, including those coding for chemokines (IL-8), adhesion molecules (endothelial leukocyte adhesion molecule, vascular cell adhesion molecule, and intercellular adhesion molecule), and cytokines (IL-1, IL-2, TNF-a, and IL-12) [48]. In the present study, we harvested renal specimens at 24 hours when KIM-1 and TNF-*α* expression was at a relative high level. The renal tissue of the sham group had almost no expression of either KIM-1 or TNF-*α*, and the nonischemic area of the SRAC group exhibited a statistically significant difference compared to the ischemic area of both the SRAC and RAC groups. These results revealed that the differences between surgical approaches play an important role in renal injury.

In conclusion, we demonstrated that precise segmental renal artery clamping (SRAC) could effectively alleviate I/R-induced renal injury by improving histopathological changes in the glomeruli, tubules, and mitochondria and attenuating creatinine, BUN, UMAB, KIM-1, and TNF-*α* levels in diabetic mice after surgery. This study suggests that the difference between surgical approaches plays an important role in renal injury and SRAC can not only effectively reduce early renal injury induced by I/R but also accelerate the recovery of renal function in diabetic mice. Thus, our data provide powerful proof that SRAC can be an ideal operative technique use in partial nephrectomies, especially for those with diabetic nephropathy and other renal insufficiencies.

## Figures and Tables

**Table 1:** Blood glucose levels and body weights of the groups.

Groups	Blood glucose levels (mg/dL)			Body weights (g)		
	Initiation of study	One week after GSE treatment	After surgical procedure	Initiation of study	One week after GSE treatment	After surgical procedure
GSE(−)/sham	29.83 ± 4.93	32.51 ± 5.38	39.01 ± 6.45	36.93 ± 2.59	40.93 ± 2.15	49.1 ± 2.57
GSE(−)/SRAC	28.96 ± 4.79	30.07 ± 2.28	35.12 ± 2.96	39.29 ± 2.06	44.39 ± 2.33	53.26 ± 2.77
GSE(−)/RAC	27.26 ± 2.6	29.6 ± 1.44	35.53 ± 1.73	35.67 ± 1.87	42.1 ± 2.19	50.53 ± 2.61
GSE(+)/sham	28.69 ± 5.71	31.77 ± 1.02	36.9 ± 2.72	33.99 ± 3.01	39.06 ± 3.43	50.8 ± 4.49
GSE(+)/SRAC	29.16 ± 2.73	31.6 ± 1.32	36.25 ± 1.55	36.87 ± 2.67	41.67 ± 3.04	49.03 ± 2.59
GSE(+)/RAC	30.84 ± 0.99	30.64 ± 1.64	37.54 ± 4.14	35.09 ± 3.31	40.91 ± 3.87	48.29 ± 4.55

## References

[1] Mir M. C., Campbell R. A., Sharma N. (2013). Parenchymal volume preservation and ischemia during partial nephrectomy: functional and volumetric analysis. *Urology*.

[2] Thompson R. H., Lane B. R., Lohse C. M. (2012). Renal function after partial nephrectomy: effect of warm ischemia relative to quantity and quality of preserved kidney. *Urology*.

[3] Nathan D. M. (2015). Diabetes: advances in diagnosis and treatment. *JAMA*.

[4] Woodrow G., Brownjohn A. M., Turney J. H. (1994). Acute renal failure in patients with type 1 diabetes mellitus. *Postgraduate Medical Journal*.

[5] Shi H., Patschan D., Epstein T., Goligorsky M. S., Winaver J. (2007). Delayed recovery of renal regional blood flow in diabetic mice subjected to acute ischemic kidney injury. *American Journal of Physiology—Renal Physiology*.

[6] Zhou S.-P., Liao W.-T., Yang L.-K., Sun L. (2013). Effects of sevoflurane pretreatment on renal Src and FAK expression in diabetic rats after renal ischemia/reperfusion injury. *Molecular and Cellular Biochemistry*.

[7] Abu-Saleh N., Awad H., Khamaisi M. (2014). Nephroprotective effects of TVP1022, a non-MAO inhibitor S-isomer of rasagiline, in an experimental model of diabetic renal ischemic injury. *American Journal of Physiology—Renal Physiology*.

[8] Melin J., Hellberg O., Funa K., Hällgren R., Larsson E., Fellström B. C. (2006). Ischemia-induced renal expression of hyaluronan and CD44 in diabetic rats. *Nephron - Experimental Nephrology*.

[9] Shao P., Qin C., Yin C. (2011). Laparoscopic partial nephrectomy with segmental renal artery clamping: technique and clinical outcomes. *European Urology*.

[10] Shao P., Tang L., Li P. (2013). Application of a vasculature model and standardization of the renal hilar approach in laparoscopic partial nephrectomy for precise segmental artery clamping. *European Urology*.

[11] Qian J., Li P., Qin C. (2015). Laparoscopic partial nephrectomy with precise segmental renal artery clamping for clinical t1b tumors. *Journal of Endourology*.

[12] Neely W. A., Turner M. D. (1959). The effect of arterial, venous, and arteriovenous occlusion on renal blood flow. *Surgery, gynecology & obstetrics*.

[13] Orvieto M. A., Zorn K. C., Mendiola F. (2007). Recovery of renal function after complete renal hilar versus artery alone clamping during open and laparoscopic surgery. *Journal of Urology*.

[14] Huang Y., Don-Wauchope A. C. (2011). The clinical utility of kidney injury molecule 1 in the prediction, diagnosis and prognosis of acute kidney injury: a systematic review. *Inflammation and Allergy—Drug Targets*.

[15] Esposito E., Cuzzocrea S. (2009). TNF-alpha as a therapeutic target in inflammatory diseases, ischemia-reperfusion injury and trauma. *Current Medicinal Chemistry*.

[16] Esposito E., Mazzon E., Muià C., Meli R., Sessa E., Cuzzocrea S. (2007). Splanchnic ischemia and reperfusion injury is reduced by genetic or pharmacological inhibition of TNF-*α*. *Journal of Leukocyte Biology*.

[17] Yonguc G. N., Dodurga Y., Adiguzel E. (2015). Grape seed extract has superior beneficial effects than vitamin E on oxidative stress and apoptosis in the hippocampus of streptozotocin induced diabetic rats. *Gene*.

[18] Okada S., Shikata K., Matsuda M. (2003). Intercellular adhesion molecule-1-deficient mice are resistant against renal injury after induction of diabetes. *Diabetes*.

[19] Pei F., Li B.-Y., Zhang Z. (2014). Beneficial effects of phlorizin on diabetic nephropathy in diabetic db/db mice. *Journal of Diabetes and Its Complications*.

[20] Kumskova E. M., Antonova O. A., Balashov S. A., Tikhaze A. K., Melkumyants A. M., Lankin V. Z. (2014). Malonyldialdehyde and glyoxal act differently on low-density lipoproteins and endotheliocytes. *Molecular and Cellular Biochemistry*.

[21] Schramm L., Weierich T., Heldbreder E., Zimmermann J., Netzer K.-O., Wanner C. (2005). Endotoxin-induced acute renal failure in rats: effects of L-arginine and nitric oxide synthase inhibition on renal function. *Journal of Nephrology*.

[22] Spek C. A., Brüggemann L. W., Borensztajn K. S. (2010). Protease-activated receptor 2 blocking peptide counteracts endotoxin-induced inflammation and coagulation and ameliorates renal fibrin deposition in a rat model of acute renal failure. *Shock*.

[23] (2002). K/DOQI clinical practice guidelines for chronic kidney disease: evaluation, classification, and stratification. *AM J KIDNEY DIS*.

[24] Idonije B. O., Festus O., Oluba O. M. (2011). Plasma glucose, creatinine and urea levels in type 2 diabetic patients attending a Nigerian teaching hospital. *Research Journal of Medical Sciences*.

[25] Vivek A., Ranjit K. S., Parduman S., Arora M. M., Somani B. L. (2004). Incidence of microalbuminuria in hypertensive patients. *Indian Journal of Medical Biochemistry*.

[26] Hasanato R. M. (2016). Diagnostic efficacy of random albumin creatinine ratio for detection of micro and macro-albuminuria in type 2 diabetes mellitus. *Saudi Medical Journal*.

[27] Sato M., Bagchi D., Tosaki A., Das D. K. (2001). Grape seed proanthocyanidin reduces cardiomyocyte apoptosis by inhibiting ischemia/reperfusion-induced activation of JNK-1 and C-JUN. *Free Radical Biology and Medicine*.

[28] Shao P., Tang L., Li P. (2012). Precise segmental renal artery clamping under the guidance of dual-source computed tomography angiography during laparoscopic partial nephrectomy. *European Urology*.

[29] Fouad A. A., Al-Mulhim A. S., Jresat I., Morsy M. A. (2013). Protective effects of captopril in diabetic rats exposed to ischemia/reperfusion renal injury. *Journal of Pharmacy and Pharmacology*.

[30] Umul M., Cal A. C., Turna B., Oktem G., Aydin H. H. (2016). Effect of complete hilar versus only renal artery clamping on renal histomorphology following ischemia/reperfusion injury in an experimental model. *European Review for Medical and Pharmacological Sciences*.

[31] Siegel N. J., Avison M. J., Reilly H. F., Alger J. R., Shulman R. G. (1983). Enhanced recovery of renal ATP with postischemic infusion of ATP-MgCl_2_ determined by 31P-NMR. *The American Journal of Physiology*.

[32] Atkinson S. J., Hosford M. A., Molitoris B. A. (2004). Mechanism of actin polymerization in cellular ATP depletion. *The Journal of Biological Chemistry*.

[33] Sharfuddin A. A., Molitoris B. A. (2011). Pathophysiology of ischemic acute kidney injury. *Nature Reviews Nephrology*.

[34] Birk A. V., Liu S., Soong Y. (2013). The mitochondrial-targeted compound SS-31 re-energizes ischemic mitochondria by interacting with cardiolipin. *Journal of the American Society of Nephrology*.

[35] Anderson S. E., Dickinson C. Z., Liu H., Cala P. M. (1996). Effects of Na-K-2Cl cotransport inhibition on myocardial Na and Ca during ischemia and reperfusion. *American Journal of Physiology - Cell Physiology*.

[36] Liu S., Soong Y., Seshan S. V., Szeto H. H. (2014). Novel cardiolipin therapeutic protects endothelial mitochondria during renal ischemia and mitigates microvascular rarefaction, inflammation, and fibrosis. *American Journal of Physiology—Renal Physiology*.

[37] Iwai T., Tanonaka K., Inoue R., Kasahara S., Kamo N., Takeo S. (2002). Mitochondrial damage during ischemia determines post-ischemic contractile dysfunction in perfused rat heart. *Journal of Molecular and Cellular Cardiology*.

[38] Kaasik A., Safiulina D., Zharkovsky A., Veksler V. (2007). Regulation of mitochondrial matrix volume. *American Journal of Physiology - Cell Physiology*.

[39] Szeto H. H., Liu S., Soong Y. (2011). Mitochondria-targeted peptide accelerates ATP recovery and reduces ischemic kidney injury. *Journal of the American Society of Nephrology*.

[40] Peng H., Mao Y., Fu X., Feng Z., Xu J. (2015). Comparison of biomarkers in rat renal ischemia-reperfusion injury. *International Journal of Clinical and Experimental Medicine*.

[41] Han W. K., Bailly V., Abichandani R., Thadhani R., Bonventre J. V. (2002). Kidney Injury Molecule-1 (KIM-1): a novel biomarker for human renal proximal tubule injury. *Kidney International*.

[42] Zhou Y., Vaidya V. S., Brown R. P. (2008). Comparison of kidney injury molecule-1 and other nephrotoxicity biomarkers in urine and kidney following acute exposure to gentamicin, mercury, and chromium. *Toxicological Sciences*.

[43] Wunnapuk K., Liu X., Gobe G. C. (2014). Kidney biomarkers in MCPA-induced acute kidney injury in rats: reduced clearance enhances early biomarker performance. *Toxicology Letters*.

[44] Vaidya V. S., Ramirez V., Ichimura T., Bobadilla N. A., Bonventre J. V. (2006). Urinary kidney injury molecule-1: a sensitive quantitative biomarker for early detection of kidney tubular injury. *American Journal of Physiology—Renal Physiology*.

[45] Speir R. W., Stallings J. D., Andrews J. M., Gelnett M. S., Brand T. C., Salgar S. K. (2015). Effects of valproic acid and dexamethasone administration on early bio-markers and gene expression profile in acute kidney ischemia-reperfusion injury in the rat. *PLoS ONE*.

[46] Tremblay J., Chen H., Peng J. (2002). Renal ischemia-reperfusion injury in the rat is prevented by a novel immune modulation therapy. *Transplantation*.

[47] Graham W. V., Wang F., Clayburgh D. R. (2006). Tumor necrosis factor-induced long myosin light chain kinase transcription is regulated by differentiation-dependent signaling events: characterization of the human long myosin light chain kinase promoter. *Journal of Biological Chemistry*.

[48] Tak P. P., Firestein G. S. (2001). NF-*κ*B: a key role in inflammatory diseases. *Journal of Clinical Investigation*.

